# FLI1 Induces Megakaryopoiesis Gene Expression Through WAS/WIP-Dependent and Independent Mechanisms; Implications for Wiskott-Aldrich Syndrome

**DOI:** 10.3389/fimmu.2021.607836

**Published:** 2021-02-26

**Authors:** Chunlin Wang, Klarke M. Sample, Babu Gajendran, Philipp Kapranov, Wuling Liu, Anling Hu, Eldad Zacksenhaus, Yanmei Li, Xiaojiang Hao, Yaacov Ben-David

**Affiliations:** ^1^State Key Laboratory for Functions and Applications of Medicinal Plants, Guizhou Medical University, Guiyang, China; ^2^The Key Laboratory of Chemistry for Natural Products of Guizhou Province and Chinese Academic of Sciences, Guiyang, China; ^3^The National Health Commission’s Key Laboratory of Immunological Pulmonary Disease, Guizhou Provincial People’s Hospital, The Affiliated Hospital of Guizhou University, Guiyang, China; ^4^School of Biomedical Sciences, Institute of Genomics, Huaqiao University, Xiamen, China; ^5^Department of Medicine, University of Toronto, Toronto, ON, Canada; ^6^Division of Advanced Diagnostics, Toronto General Research Institute—University Health Network, Toronto, ON, Canada

**Keywords:** Wiskott–Aldrich Syndrome, megakaryopoiesis, microthrombocytopenia, FLI1, immunodeficiency, WASP, WIP, N-WASP

## Abstract

Wiskott–Aldrich Syndrome, WAS/WAVE, is a rare, X-linked immune-deficiency disease caused by mutations in the *WAS* gene, which together with its homolog, N-*WASP*, regulates actin cytoskeleton remodeling and cell motility. WAS patients suffer from microthrombocytopenia, characterized by a diminished number and size of platelets, though the underlying mechanism is not fully understood. Here, we identified FLI1 as a direct transcriptional regulator of *WAS* and its binding partner *WIP*. Depletion of either *WAS* or *WIP* in human erythroleukemic cells accelerated cell proliferation, suggesting tumor suppressor function of both genes in leukemia. Depletion of *WAS/WIP* also led to a significant reduction in the percentage of CD41 and CD61 positive cells, which mark committed megakaryocytes. RNAseq analysis revealed common changes in megakaryocytic gene expression following FLI1 or WASP knockdown. However, in contrast to FLI1, WASP depletion did not alter expression of late-stage platelet-inducing genes. N-WASP was not regulated by FLI1, yet its silencing also reduced the percentage of CD41+ and CD61+ megakaryocytes. Moreover, combined knockdown of WASP and N-WASP further suppressed megakaryocyte differentiation, indicating a major cooperation of these related genes in controlling megakaryocytic cell fate. However, unlike WASP/WIP, N-WASP loss suppressed leukemic cell proliferation. WASP, WIP and N-WASP depletion led to induction of FLI1 expression, mediated by GATA1, and this may mitigate the severity of platelet deficiency in WAS patients. Together, these results uncover a crucial role for FLI1 in megakaryocyte differentiation, implicating this transcription factor in regulating microthrombocytopenia associated with Wiskott–Aldrich syndrome.

## Introduction

Wiskott–Aldrich Syndrome (WAS) is a rare X-linked recessive disease, that affects 1–10 men per million (1). WAS patients exhibit both cellular and humoral immunodeficiency, eczema, high susceptibility to infections, microthrombocytopenia (low platelet count), increased risk of autoimmune disorders and lymphomas (2). Deletions or mutations in *WAS* affects the expression and function of its protein (WASP), with direct correlation between the impact on its function and severity of the disease (3, 4).

WASP is expressed in hematopoietic cells (1). Its encoded protein has multi-domains through which it interacts with several partners to execute diverse functions (2). WASP regulates the actin cytoskeleton thereby affecting amoeboid motility, endocytosis, and pathogen invasion (5). Following phosphorylation by upstream signaling events, WASP binds to the Actin Related Protein (ARP) 2/3 complex. Activated ARP2/3 then induces nucleation of actin filaments, formation of a branching network of actin at the plasma membrane (6–9), leading to motility, endocytosis, changes in cell shape, and phagocytosis (10).

FLI1, a critical transcription factor (TF) in hematopoiesis, is overexpressed and drives leukemogenesis and other malignancies (11). FLI1 knockout mice exhibit embryonic lethality due to loss of vascular integrity and defects in megakaryopoiesis (12, 13). In man, hemizygous loss of FLI1 results in platelet deficiency and is the cause of Paris-Trousseau/Jacobsen thrombocytopenia (14–16). Conversely, drug-mediated activation of FLI1 by phorbol ester compound 12-O-Tetradecanoylphorbol-13-acetate (TPA) induces megakaryopoiesis in leukemic cells, which is associated with suppression of proliferation, cell attachment, polyploidy, and induction of the megakaryocytic genes (17). However, the underlying mechanism of FLI1-mediated megakaryopoiesis is unknown, and no connection to WAS has ever been reported. In this study, we identified a direct correlation between expression of FLI1, WAS, and its related binding protein WIP (also known as WIPF1) in multiple leukemic cell lines. We show that the *WAS* and *WIP* promoters have binding sites for FLI1 and are transcriptionally activated by this TF. In accordance with observations that FLI1 loss in hematopoietic cells blocks megakaryocytic differentiation (12, 13), shRNA-mediated knockdown of *WAS/WIP* also suppressed this maturation process. FLI1 did not regulate the neural variant of WAS, N-WASP (also known as WASL), which similarly to WAS plays a critical role in megakaryocytic differentiation (18). Yet, knockdown of N-WASP suppressed megakaryocytic differentiation. While WAS/WIP behaves as tumor suppressor genes, N-WASP accelerated leukemia progression. Together, our results reveal a role for FLI1 in regulating WAS/WIP and megakaryopoiesis, with direct mechanistic and therapeutic implications for microthrombocytopenia in WAS patients.

## Materials and Methods

### Cell Lines and Tissue Culture Assays

Erythroleukemia cell lines (human HEL [ATCC-TIB-180], HEK293T [ATCC-CRL3216] and K562 [ATCC-CCL-243]; mouse KH16, CB7, CB3, HB2.22 and DP17 erythroleukemias (19–21) tested negative for mycoplasma, were cultured and maintained in Dulbecco’s Modified Eagle Medium supplemented with 5% fetal bovine serum (HyClone, GE Healthcare).

For drug treatment, the HEL cells were treated with the indicated concentrations of A661, A665, and TPA (22) and 24 h later were used for microscopic examination, flow cytometry or western blot analysis. The Anti-FLI1 compounds were dissolved to a stock solution of 2 mM in dimethyl sulfoxide (DMSO), diluted to concentrations indicated in the figures/figure legends. DMSO was also used as a vehicle control.

The K562-fli1 inducible cell line was generated as previously described (17). To induce FLI1, 3 × 10^6^ cells were treated for 48 h with 10 nM of doxycycline (Cat. no. D8960-5g, Solarbio, Beijing, China) and used in the experiments described below.

### Promoter Cloning, Transfection and Luciferase Assays

**Figure 2:**
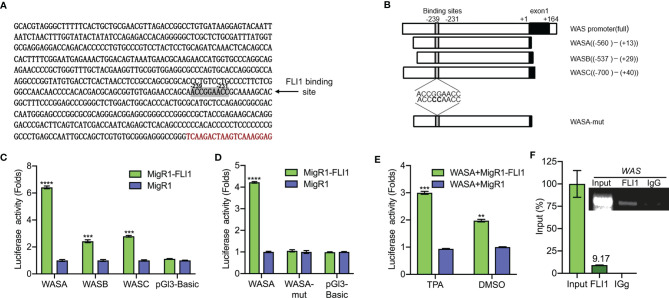
FLI1 regulates the *WAS* promoter in erythroleukemic cells. **(A)** Sequence of the *WAS* promoter highlighting the putative FLI1 binding site. **(B)** Promoter regions of the *WAS* gene cloned upstream to the pGL3 luciferase reported plasmid (Left panel). WASA-mut shows the position of mutation within the WASA promoter. **(C)** Transfection of MigR1-FLI1 expression vector with the *WAS* promoters (WASA, WASB, WASC) into HEK-293T cells resulted in significantly higher luciferase activity relative to the control MigR1-vector transfected cells. **(D)** ACCGGAACC to ACCCCAACC mutations within the FLI1 binding site in the WASA promoter suppressed luciferase activity. **(E)** Treatment of HEL cells with the FLI1 activator Phorbol myristate acetate (TPA; 1 μM; 24 h) resulted in significant induction of FLI1 and WASP expression. **(F)** ChIP analysis of the *WAS* promoter for binding to FLI1 in HEL cells, followed by Q-PCR (Lower panel). Percentage of immunoprecipitation relative to input (9.17%) is indicated. Upper panel shows the gel image of the immunoprecipitated PCR-amplified band relative to input. ***P<0.001 and ****P<0.0001.

For promoter cloning, various DNA regions of *WAS* (for details see **Figure 2B** and **Supplementary Table 1**) were amplified by PCR and after isolation, the DNA fragment was cloned into the luciferase reporter vector PGL3 (Promega), as previously described (23). The primers used for cloning are listed in **Supplementary Table 3**. The 1 μg of the *WAS* promoter was co-transfected with either MigR1 (1 μg) or MigR1-FLI1 (1 μg) into HEK-293T cells using Lipofectamine 2000 (Life Technologies; Thermo Fisher Scientific), following the manufacturer’s protocol. Renilla luciferase was used in the transfection as an internal control for the transfection efficiency, according to the manufacturer’s recommendations (Promega, Madison, Wisconsin, USA). The transfected cells were then seeded onto into 96-well plates (6 × 10^3^ cells/well) and luciferase activity was determined, as previously described (23).

The shFLI1 (22), shWASP, shWIP, shN-WASP and scrambled constructs were generated by synthesizing and cloning shRNA of WASP, WIP, N-WASP, and scrambled DNA into the BcuI sites of PLent-GFP plasmid (Vigene Bioscience, Rockville, MD, USA). For lentivirus production, shRNA expression plasmids (12 μg) and packaging plasmids psPAX2 (6 μg) and pMD2.G (12 μg) (a gift from Didier Trono, Addgene plasmid #12259 and #12260) were co-transfected into HEK293T cells, using Lipofectamine 2000, as described (22, 24). 48 h post transfection, the supernatant was collected and used to transduce HEL (1 × 10^6^) cells. The medium was changed 24 h post transduction and positive cells were selected for using medium containing puromycin (5 μg/ml) [Solarbio]. The sequences for the shRNAs and their controls were shown in **Supplementary Table 2**. SiRNAs for N-WASP and GATA1 were purchased from GenePharma, China. SiRNAs were transfected into shWASP-HEL cells alongside a scrambled control, as previously described (22). The sequences of the siRNAs are listed in **Supplementary Table 2**.

### Chromatin Immunoprecipitation Analysis

**Figure 3:**
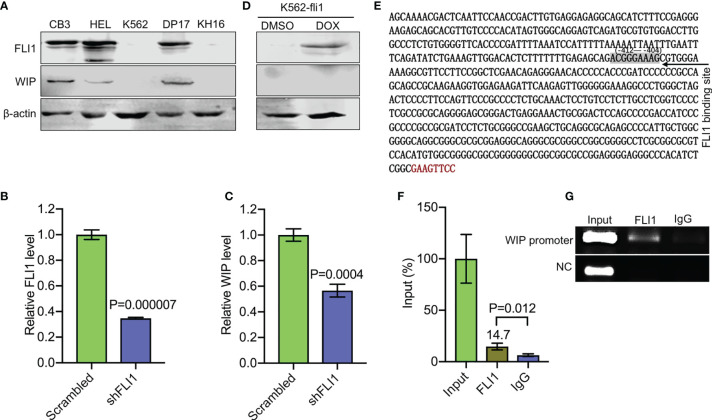
FLI1 regulates the *WIP* gene in erythroleukemic cells. **(A)** Positive correlation between FLI1 and WIP protein levels in indicated cell lines. **(B, C)** Knockdown of *FLI1* by shFLI1 in HEL cells **(B)** reduces expression of *WIP* **(C)**, as determined by Q-RT-PCR. **(D)** Expression of FLI1 and WIP in K562-fli1 cells after addition of doxycycline. **(E)** Sequence of the *WIP* promoter with the position of the FLI1 binding site (positions −412 to −404). **(F)** ChIP analysis of the *WIP* promoter region for its binding to FLI1 followed by Q-RT-PCR. **(G)** Gel images of the immunoprecipitated PCR-amplified band surrounding the FLI1 binding region of the *WIP* promoter.

ChIP analysis was performed, as previously described (22). In brief, HEL cells were crosslinked with formaldehyde and resuspended in lysis buffer Magna ChIP A/G kits (Cat. no. 17-10085, Millipore [Sigma-Aldrich, China). Fixed cells were then sonicated using a Sonics Vibra VCX150 (Ningbo Scientz Biotechnology, Hangzhou, China). At this stage, a chromatin aliquot was removed for the input control. Protein G sepharose beads were added to the isolated chromatin and incubated for 1 h at room temperature. The immunoprecipitations were performed overnight at 4°C with 1 μg of FLI1 antibody (ab15289, Abcam, Cambridge, UK) or a non-specific normal mouse immunoglobulin G (IgG) antibody (Millipore). The precipitates were subsequently washed and reverse crosslinked, using the instructions provided with the Magna ChIP G kits (Millipore). Precipitated chromatin was then incubated with proteinase K at 56°C for 2 h, DNA-purified with phenol chloroform extraction and resuspended in TE buffer. Q-RT-PCR was performed to determine the level of FLI1 binding within the *WAS* and *WIP* promoter regions (for *WAS* position −329 to −231 and for *WIP* position −412 to −404). The sequences of the ChIP primers are presented in **Supplementary Table 3**. The percentage of input was calculated by Q-RT-PCR based upon the intensity of the amplified FLI1 DNA divided by the amplified input DNA. Amplified DNA was also resolved on a 2% agarose gel and illustrated in **Figure 2F** (right panel) and **Figure 3G**. ChIP was performed at least in three independent experiments.

### RNA Preparation, Q-RT-PCR

Total RNA was isolated from cultured HEL cells using TRIzol reagent (Life Technologies; Thermo Fisher Scientific, USA), according to the manufacturer’s protocol. RNA was quantified using a NanoDrop 2000 spectrophotometer (Thermo Fisher Scientific). To synthesize cDNA, reverse transcription reaction was performed using the PrimeScript RT Reagent kit (Takara Bio, Beijing, China). Q-RT-PCR was performed on these cDNAs using FastStart Universal SYBR-Green Master (Roche, Shanghai, China) and the Step One Plus Real-time PCR system [Applied Biosystems/Thermo Fisher Scientific (24)]. *β*-actin level was used to normalize expression. The primer sequences are listed in **Supplementary Table 3**. Three biological replicates were used for all the Q-RT-PCRs, each in triplicate (n = 3).

### Western Blot Analysis

Western blotting was performed, as previously described (22, 23). The following antibodies were used: Polyclonal rabbit antibodies for FLI1 (Cat. no. ab133485), ERK (ab184699) and FLI1ChIP grade (Cat. no. ab15289) were all purchased from Abcam; the WIP (Cat. no. PA5-51995) antibody was obtained from Invitrogen (Invitrogen; the phospho-ERK (Cat. no.9101S) antibody was obtained from Cell Signaling Technology (CST, Danvers, MA01923); the GAPDH (Cat. no. G9545) antibody was obtained from Sigma Aldrich; WASP (Cat. no. 10879-1-AP), N-WASP (Cat. no. 14306-1-AP), GATA1 (Cat. no. 10917-2-AP), *β*-actin antibodies (Cat. no. 20536 1 AP) were obtained from Proto Technology (Proteintech, Bucuresti, Romania); goat anti-mouse and goat anti-rabbit HRP conjugated antibodies were obtained from Cell Signaling Technology (Cat. nos. 5470s and 5151s, respectively). Antibody dilution was conducted according to the manufacturer’s instructions. The Odyssey system (LI COR Biosciences) and Bio were used to image proteins in western blot analysis.

The inhibitor of N-WASP (Wiskostatin) was obtained from Cayman Chemica (Cat. no. 15047-10). The development of our specific FLI1 inhibitory compounds A661 and A665 has previously been described (22).

### Flow Cytometric Analysis

Immunofluorescence staining was conducted to detect erythroid and megakaryocytic cells, as previously described (22, 24). In brief, 1 × 10^6^ cells were stained with APC- and FITC-conjugated antibodies for 40 min at RT. Cells were then washed twice and resuspended in 500 μl of phosphate buffered saline and used for flow analysis. The following primary antibodies were used: human CD41a-FITC (Cat. no. 555466), human CD41a-APC (Cat. no. 559777), human CD61-APC (Cat. no. 564174), human CD71-APC (Cat. no. 551374), human CD71-FITC (Cat. no. 555536), human CD235a-APC (Cat. no. 551336) were all purchased from BD Biosciences (BD Biosciences, NJ, USA). Flow cytometry was performed using a NovoCyte flow cytometer (ACEC Biosciences Inc, CA, USA) and Novo-express software.

The following gating strategies were used: FSC-A/SSC-A plots were used to separate live cells from debris. Erythroid cells were differentiated using a scatter/anti-CD71+ and a scatter/anti-CD235a+ gate from unstained control, respectively. Megakaryocytes were differentiated using a scatter/anti-CD41a+ and a scatter/anti-CD61+ gate from unstained control, respectively. Count/anti-CD71+, count/anti-CD235a+, count/anti-CD41a+, and count anti-CD61+ in histograms present the expression of these markers.

### CCLE Data Analysis

Data from the CCLE (Cancer Cell Line Encyclopedia) was downloaded [Broad Institute, 2019 (25);] for FLI1, WIP, WAS, and N-WASP from cBioportal in the form of mRNA expression z-Scores. GraphPad Prism 8 was used to produce the correlation analysis (two-tailed Spearman’s Rho) and to compare the expression of the aforementioned genes between the Haematopoietic and Lymphoid cells and other cell types (two-tailed Mann–Whitney U test).

### RNAseq and Heatmap Analysis

The RNA sequencing was performed using shFLI1 and shWAS HEL cells RNA by The Beijing Genomics Institute (BGI; Wuhan, China). The RNAseq data was mapped using HISAT2 and differential expression analysis was conducted with cufflinks. A list of 74 unique megakaryocyte related genes was obtained by collating human genes contained within the following gene ontology terms: negative regulation of megakaryocyte differentiation (GO:0045653, five genes), regulation of megakaryocyte differentiation (GO:0045652, 61 genes), positive regulation of megakaryocyte differentiation (GO:0045654, 10 genes), megakaryocyte differentiation (GO:0030219, 87 genes). Those genes with only trace expression (FPKM below 20 in all three conditions) were excluded leaving a total of 43 genes. An additional list of 25 unique genes from the platelet formation (GO:0030220) gene ontology term was obtained, of which 20 genes had an above trace level of expression (using the aforementioned definition). Hierarchical gene clustering was performed with Biovinci (version 1.1.5) on the curated gene groups using Ward’s minimum variance and Euclidean distance prior to presenting the data using heatmaps. List of these genes is shown in **Supplementary Tables 4–6**.

The original contributions presented in the study are publicly available. This data can be found here: https://www.ncbi.nlm.nih.gov/bioproject/?term=PRJNA682304

### Statistical Analysis

Additional statistical analyses were carried out using the two-tailed Student t-test with significance considered at by *P<0.05, **P<0.01, ***P<0.001 and ****P<0.0001, or by one-way ANOVA with Tukey’s *post-hoc* test, using Origin 3.5 software (Microcal Software). The results were expressed as the means ± standard deviation from at least three independent experiments.

## Results

### FLI1 Expression Correlates With WAS Expression in Leukemic Cells

**Figure 1:**
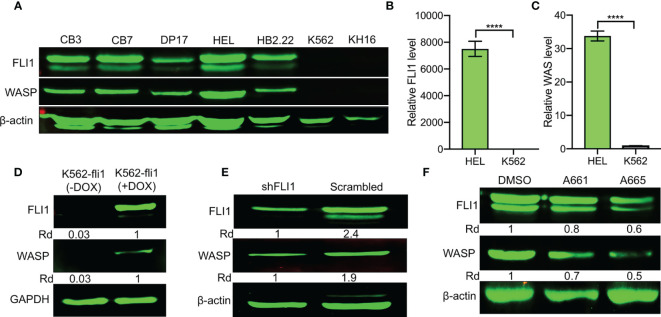
Positive correlation between FLI1 and WASP expression in erythroleukemia cell lines. **(A)** Relative expression of FLI1 and WASP in indicated cell lines. *β*-actin was used as loading control. **(B, C)** Relative transcriptional expression of *FLI1* **(B)** and *WAS* **(C)**, determined by Q-RT-PCR, in HEL and K562 cells. ****P < 0.0001. **(D)** Relative expression in FLI1 and WASP proteins in inducible K562-fli1 cells after addition of Doxycycline. **(E)** Knockdown of *FLI1* by shFLI1 in HEL cells blocks both FLI1 and WASP expression. **(F)** Treatment of HEL cells with anti-FLI1 compounds A661 and A665 (3 μM) for 24 h decreased expression of both FLI1 and WASP.

FLI1 protein is expressed in erythroleukemia cell lines CB7, CB3, HEL, DP17-17, and HB2.22, but absent in K562 and KH16 (**Figure 1A**). Remarkably, WASP was readily detected in all the cells that expressed FLI1 but not in the FLI1-negative lines (**Figure 1A**). Q-RT-PCR further demonstrated transcriptional correlation between *FLI1* and *WAS* in HEL and K562 cells (**Figures 1B, C**). These results suggested that FLI1 may transcriptionally regulate the *WAS* promoter.

To investigate this possibility, WASP expression was assessed in an inducible K562 cell system (K562-fli1), in which endogenous FLI1 is not detectable but could be induced by doxycycline (16). FLI1 induction resulted in the appearance of WASP protein (**Figure 1D**). Conversely, *FLI1* knocked-down in HEL cells *via* shRNA (shFLI1) led to reduced WASP expression (**Figure 1E**). Moreover, treatment of HEL cells with the FLI1-specific inhibitors A661 or A665 (22), diminished both FLI1 and WASP expression (**Figure 1F**). These results confirmed the correlation between FLI1 and *WAS* expression in leukemic cells and further suggested that FLI1 is an upstream transcriptional regulator of *WAS*.

### FLI1 Binds the *WAS* Promoter and Regulates Its Transcription

An analysis of the human *WAS* promoter revealed a putative FLI1 binding site (ACCGGAACC) at positions −239 to −231 relative to the transcription start site (**Figure 2A**). To determine whether FLI1 regulates the *WAP* promoter through this site, a series of DNA fragments spanning the human *WAS* promoter (WASA-WASC) were subcloned upstream of a luciferase reporter plasmid (PGL-3; **Figure 2B** and **Supplementary Table 1**). The putative FLI1 binding site was also mutated from ACCGGAACC to ACCCCAACC, yielding WASA-Mut (**Figure 2B**). These promoter constructs were transiently transfected into HEK-293T cells with a FLI1 expression vector (MigR1-FLI1) or control vector (MigR1), and transcriptional activation was examined by luciferase assays. FLI1 significantly increased luciferase activity of the WASA-WASC expression vectors relative to the control; this induction was completely abolished by the CC to GG mutation in the FLI1-consensus site (**Figures 2C, D**, respectively). We have previously shown that phorbol-ester (TPA) is a strong inducer of FLI1 transcriptional activity, mediated through phosphorylation of PKC*δ* (17). Cells transfected with WASA plus MigR1-FLI1 or WASA plus MigR1 were treated with TPA (1 μM) or vehicle control (DMSO) for 24 h. The cells treated with the FLI1 agonist TPA exhibited a greater increase in *WAS* promoter activity compared to the control, demonstrating that FLI1 transcriptionally regulates the *WAS* promoter (**Figure 2E**).

To further confirm regulation of the *WAS* promoter by FLI1, Chromatin immunoprecipitation (ChIP) assays were carried out in HEL cells, which express high levels of WAS/FLI1 (**Figure 1A**) and primers that flank the FLI1-binding site in the *WAS* promoter. ChIP with FLI1 but not control IgG antibodies revealed robust binding (**Figure 2F**), demonstrating direct recruitment of FLI1 to the *WAS* promoter.

WASP/N-WASP interacting protein 1 (WIP) binds WASP and N-WSAP to increase actin polymerization, the primary function of these factors (5, 26, 27). Knockdown of WIP in cells has previously been shown to decrease WASP expression due to reduce protein stability (26, 28). Interestingly, using immunoblot analysis, we observed a positive correlation between FLI1 and WIP expression in the erythroleukemia cell lines CB3, HEL, and DP-17, which express FLI1, but not in K562 and KH16, which lack FLI1 (**Figure 3A**). ShRNA-mediated knockdown of *FLI1* (shFLI1) in HEL cells resulted in lower expression of WIP (**Figures 3B, C****)**. However, expression of FLI1 failed to induce WIP in the inducible K562-fli1 cells (**Figure 3D**). This suggests FLI1 is necessary but not sufficient for WIP expression.

Consistent with this possibility, we identified a potential binding site for FLI1 (ACGGGAAAG at positions −412 to −404) in the promoter region of the human *WIP* gene (**Figure 3E**). A FLI1 ChIP analysis precipitated a band surrounding this binding site, which was not precipitated by control IgG (**Figures 3F, G**), thus confirming that *WIP* is a direct target of FLI1.

### FLI1 Induces Expression of Megakaryocytic Genes Through WASP and Its Partner WIP

**Figure 4:**
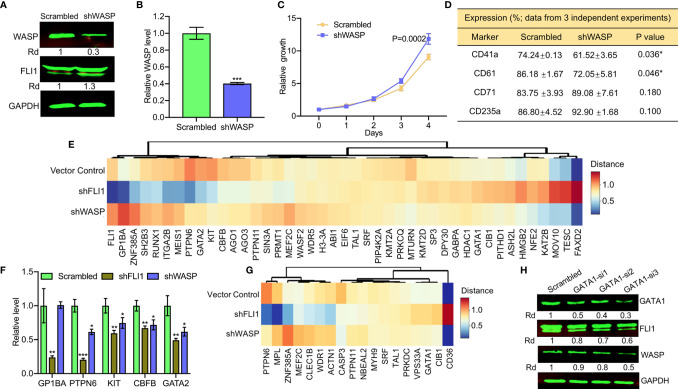
ShRNA-mediated depletion of WAS in HEL cells reduces the percentage of CD41a and CD61 positive cells. **(A, B)** ShRNA mediated depletion of WASP in HEL cells measured by western blot **(A)** or Q-RT-PCR **(B)**. Scrambled-transfected cells were used as a negative control. **(C)** shWASP-HEL cells exhibit accelerated proliferation compared to control scrambled-shRNA cells. **(D)** Flow cytometry analysis of shWASP-HEL and scrambled-HEL cells for expression of the indicated megakaryocytic (CD41a and CD61) and erythroid (CD71 and CD235a) cell surface markers in three independent experiments. **(E)** Expression of megakaryocytic genes following shFLI1, shWASP and control shRNA is presented as a heatmap. **(F)** Expression of indicated megakaryocytic genes in *FLI1* and *WASP* knockdown HEL cells relative to scrambled control, by Q-RT-PCR. **(G)** Heatmap of shFLI1, shWASP, and control for expression of platelet forming genes. **(H)** Knockdown of GATA1 by siRNA (GATA1-si1-3) in shWASP cells resulted in downregulation of FLI1 and further reduction in WASP expression. *P<0.05, **P<0.01, ***P<0.001.

Fli1−/− and Was−/− null mice exhibit robust or mild megakaryocyte defects and platelet deficiency, respectively (12, 13, 29). Therefore, we investigated whether FLI1 controls megakaryopoiesis *via* WASP and WIP. To mimic the Wiskott–Aldrish phenotype (3), bi-potential erythroid/megakaryocytic HEL progenitor cells (17) were transfected with an shRNA-WASP expression vector (shWASP), leading to reduced expression of WASP and its transcript compared to scrambled shRNA control (**Figures 4A, B**). Reduced WASP expression significantly increased proliferation in culture (**Figure 4C**), consistent with a recent report demonstrating a tumor suppressor role for this gene in T cell lymphoma (30). The WASP-knocked-down cells were then examined for expression of megakaryocytic (CD41a/CD61) and erythroid (CD71/CD235a) markers. WASP knockdown significantly reduced percentage of megakaryocytic CD41a and CD61 expressing cells and their expression intensity relative to scrambled shRNA control, but there was no difference in the percentage of erythroid CD71 and CD235a expressing cells and their intensity (**Supplementary Figure 1** and **Figure 4D**).

To further examine the contribution of FLI1 and WASP to megakaryocyte development, we performed RNAseq analysis on RNA isolated from shFLI1-HEL *versus* shWASP-HEL cells and controls. Specifically, we compared the expression of 43 megakaryocytic specific genes (see *Materials and Methods*; **Supplementary Table 4**), using a cluster analysis (**Figure 4E**). FLI1 appeared to decrease the expression of GP1BA, GATA2, RUNX1, which are known to be regulated by this TF (11, 31) and alter expression of other genes not yet known to be regulated by FLI1 (**Figure 4E**). Only a few genes including KIT, CBFB, PTPN6, and GATA2 had commonly decreased expression following the knockdown of FLI1 or WASP. The effect of knocking down FLI1 and WASP on these genes was confirmed by Q-RT-PCR (**Figure 4F**). In the late megakaryopoiesis stage, platelet formation, while loss of FLI1 strongly increased the expression of CD36 and decreased the expression of WDR1, ZNF385A, ACTN1, PTPN6, and MPL (**Figure 4G**), only CASP3 was moderately affected in both shFLI1-HEL and shWASP-HEL cells (**Supplementary Table 5** and **Supplementary Figure 2**). This result suggested a limited role for WAS in platelet formation.

Interestingly, loss of WASP significantly increased FLI1 protein (**Figure 4A**) and RNA (**Figure 4E**) expression. A previous study implicated GATA1 as a major regulator of the FLI1 promoter (32). Since GATA1 expression was induced in shWASP cells (**Supplementary Table 5**), we examined if FLI1 was upregulated in these cells through GATA1. Three siRNAs (GATA-si1-3) were designed to reduce the expression of GATA1 in shWASP cells in which GATA1-si1 showed the highest ability efficacy (**Figure 4H**). Downregulation of GATA1 resulted in lower expression of FLI1 and WASP in shWASP cells in western blot (**Figure 4H**) and Q-RT-PCR (**Supplementary Figure 3**). These results indicate that while FLI1 regulates megakaryocytic differentiation in part through WASP/WIP, the WASP mutation through upregulation of GATA1 may compensate this maturation process by upregulating FLI1. This feedback loop between WAS and FLI1 may influence the severity of megakaryopoiesis in WAS patients and implicates FLI1 in this disease.

The aforementioned RNAseq analysis also revealed a drastic expression reduction for several non-megakaryocytic genes including HDC, TPSAB1, CAPG, DHRS2, and PFDN6 following either shFLI1 or shWASP knockdown (**Supplementary Table 6**). These genes are involved in a variety of functions including histamine biosynthesis, immunity, inflammation, chromatin regulation, tumorigenesis, and others (33–37) and may affect other non-megakaryocytic phenotypes seen in WAS patients.

**Figure 5:**
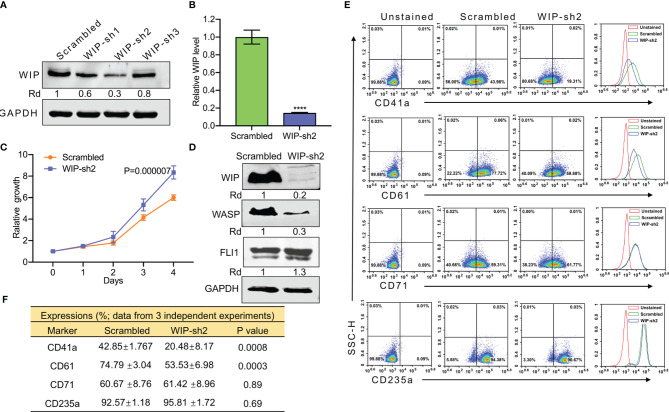
ShRNA mediated knockdown of WIP suppresses the percentage of CD41a and CD61 expressing HEL cells. **(A)** Depletion of *WIP* in HEL cells using three shRNAs (shRNA1-shRNA3). **(B)** Knockdown of *WIP* in HEL cells using shRNA2 (WIP-sh2), as determined by Q-RT-PCR. **(C)** Knockdown of WIP in HEL cells (WIPF-sh2) accelerated proliferation when compared to scrambled-transfected HEL cells. **(D)** Western blot of WIP-sh2 and control cells for expression of WIP, FLI1 and WASP. **(E)** Flow cytometry analysis of WIP-sh2 and control scrambled cells for expression of indicated megakaryocytic and erythroid markers. The unstained identical plots (left most panels) were used as negative control. **(F)** Summary and statistics of flow cytometry experiments (n = 3). ****P<0.0001.

Knockdown of WIP using shRNA (Wip-sh2) in HEL cells (**Figures 5A, B**) increased proliferation (**Figure 5C**). As expected, knockdown of WIP resulted in significant downregulation of WASP due to loss of protein stability (**Figure 5D**). WIP knockdown, similar to WASP downregulation (**Figure 4A**), markedly increased FLI1 expression (**Figure 5D**). Depletion of WIP reduced the percentage of HEL cells expressing CD41a and CD61 but had no effect on the number of CD71 or CD235a expressing cells (**Figures 5E, F**).

**Figure 6:**
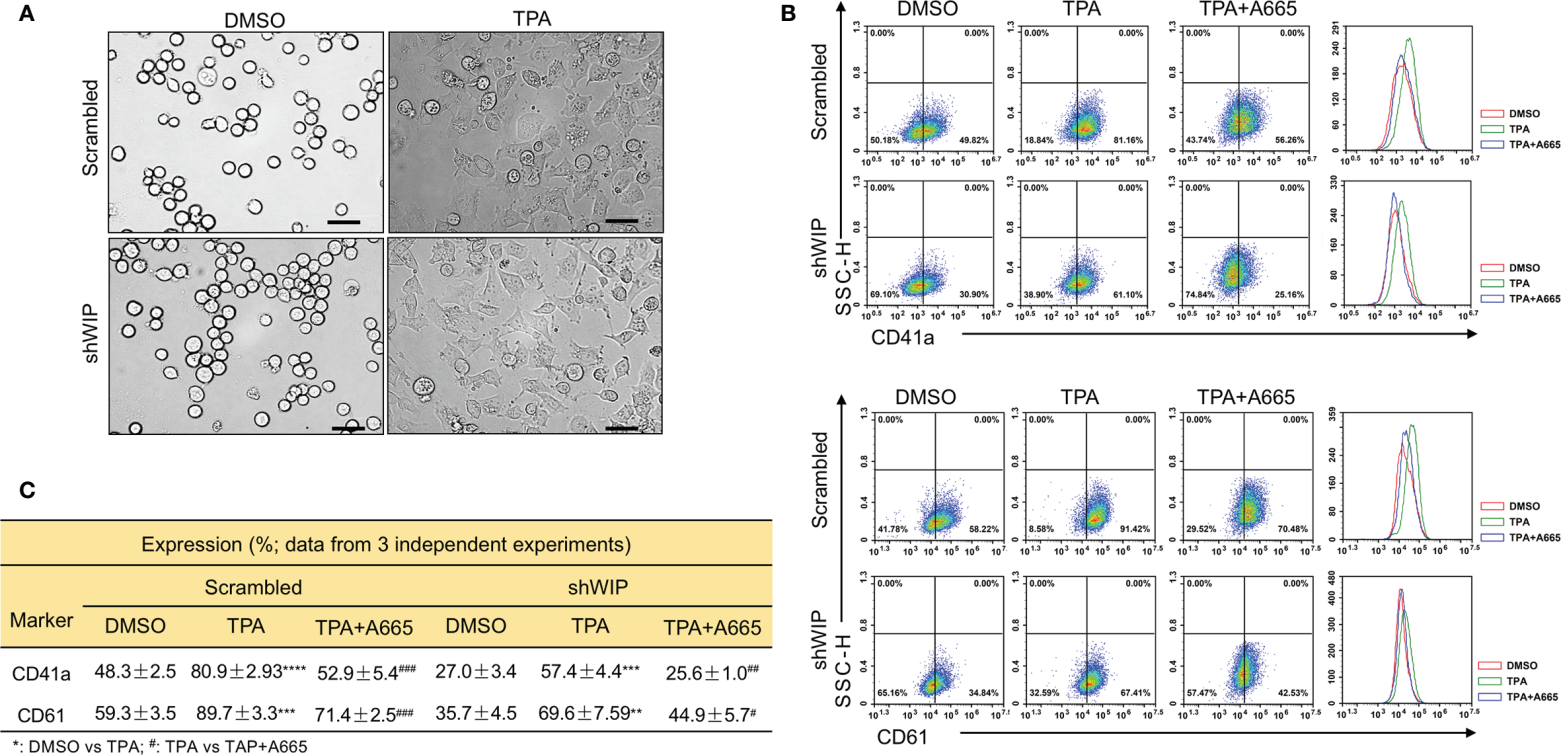
Drug mediated inhibition of FLI1 suppresses megakaryocytic differentiation induced by TPA. **(A)** Micrographs showing TPA increases the attachment and appearance of large multi-nuclear cells in HEL cells, 24 h after drug treatment (magnification ×40, scale bars, 50 μm). **(B)** Induction of megakaryocytic markers CD41a and CD61 by TPA in shWIP and scrambled control cells can be reversed by treatment with the anti-FLI1 compound A665. **(C)** Summary and statistics for the flow cytometry experiments (n = 3).

Previously, we reported on megakaryocytic differentiation of HEL cells by TPA that was mediated through activation of FLI1 (17). This TPA-induced megakaryocytic differentiation was associated with morphological changes including cell attachment, enlargement, and polyploidy (17). Interestingly, when shWIP and scrambled control cells were treated with TPA for 24 h, the DMSO and TPA-treated cells showed similar morphological changes resembling megakaryocytic differentiation (**Figure 6A**). By flow cytometry, the percentage of CD42a and CD41a positive cells was observed as being significantly lower in shWIP *vs* scrambled cells. Treatment with TPA increased at similar rate the percentage of CD41a and CD61 cells in both shWIP and scrambled cells (**Figures 6B, C**). However, when shWIP-TPA and scrambled-TPA cells were treated with the anti-FLI1 compound A665, the percentage of CD41a and CD61 positive cells decreased in both groups almost to the level of the control DMSO treated cells (**Figures 6B, C**), indicating FLI1-dependent megakaryocytic differentiation by TPA. Since the level of FLI1 is markedly increased in shWIP cells (**Figure 5D**) and shWASP (**Figure 4A**), higher expression of this TF likely acts as a compensatory mechanism to further increase the percentage of CD41a and CD61 expressing cells in shWIP-TPA cells. Overall, these results suggest that FLI1 regulates megakaryocytic differentiation in part through WASP and WIP. In support of these observations, a CCLE (Cancer Cell Line Encyclopedia) analysis predicted high expression of WAS and WIP in FLI1-positive hematological malignancies (**Supplementary Figures 4A, B** and **E, F**).

### Regulation of Megakaryopoiesis by the *WAS* Related Gene (N-*WASP*)

Given a previous report implicating N-WASP in megakaryopoiesis (18), we next asked whether the *N-WASP* promoter is also regulated by FLI1. A potential FLI1 binding site is identified at positions −129 to −137 (**Supplementary Figure 5**). However, FLI1 depletion in shFLI1-HEL cells had no effect on N-*WASP* mRNA levels (**Supplementary Figures 6A, B**). In addition, while *WASP* expression was induced following doxycycline induction of FLI1 in K562-fli1 cells, there was only a negligible effect on N-*WASP* mRNA (**Supplementary Figure 6C**) and protein (**Supplementary Figure 6D**) levels. WASP depletion in shWAS-HEL cells also had no detectable effect on N-WASP expression (**Supplementary Figure 6E**). Thus, unlike WAS and WIP, FLI1 does not seem to regulate the *N-WASP* promoter. Accordingly, CCLE analysis revealed no correlation between N-WASP expression and FLI1- (**Supplementary Figures 4C, E, F**) or N-WASP and WAS- (**Supplementary Figures 4D–F**) positive hematological malignancies.

### N-WASP Depletion in Pre-Leukemic Cells Reduces the Percentage of CD41 and CD61 Megakaryocytic Markers

**Figure 7:**
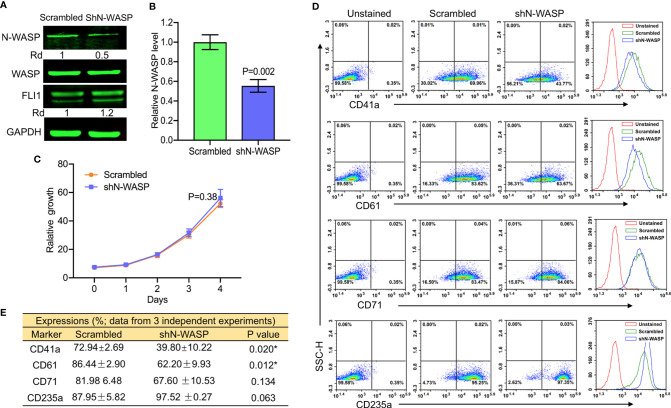
ShRNA-mediated knockdown of N-WASP in HEL cells reduces the percentage of CD41a and CD61 positive cells. **(A)** Western blotting demonstrated lower expression of N-WASP in shN-WASP-HEL compared to scrambled-HEL cells. **(B)** Knockdown of N-*WASP* in shN-WASP-HEL cells by Q-RT-PCR. **(C)** Proliferation rate of shN-WASP-HEL relative to control scrambled-HEL cells. **(D)** Flow cytometry analysis of shN-WASP-HEL and corresponding scrambled-HEL cells for expression of indicated megakaryocytic and erythroid markers. The unstained identical plots (left most panels) were used as negative control. **(E)** Summary of the flow data for experiments (n = 3).

To further investigate the effect of N-WASP on megakaryopoiesis, we silenced its expression by shRNA (shN-WASP) in HEL cells (**Figures 7A, B**). Similar to the effect of WASP/WIP depletion (**Figure 4A** and **Figure 5D**), silencing of N-WASP was upregulated FLI1 expression (**Figure 7A**). However, in contrast to WASP, N-WASP depletion resulted in only marginal changes in cell proliferation (**Figure 7C**). Nonetheless, N-WASP loss significantly decreased the percentage of CD41a and CD61 expressing cells relative to control transfected cells (**Figure 7D**). While the percentage of shN-WASP-HEL cells expressing the erythroid marker CD71 decreased; the number of more committed CD235a-positive erythroid cells was significantly increased (**Figures 7D, E**).

**Figure 8:**
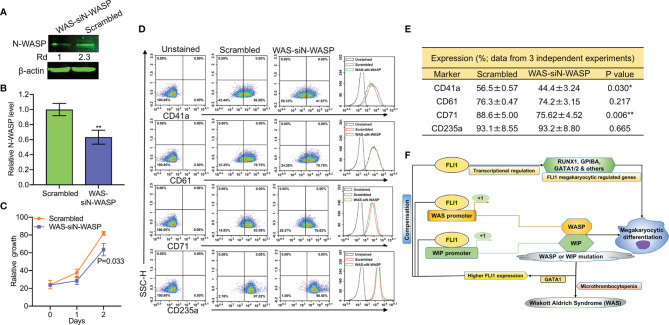
SiRNA-mediated knockdown of N-WASP in shWASP-HEL cells further reduces percentage of CD41a and CD61 expressing cells. **(A)** N-WASP knockdown in shWASP-HEL cells (shWAS-siN-WASP) compared to control scrambled-HEL cells. **(B)** Expression analysis of N-*WASP* depletion following transient knockdown *via* si1 in shWASP-HEL cells, using Q-RT-PCR. **(C)** Proliferation rate of WAS-siN-WASP-HEL cells *versus* control scrambled-HEL cells. **(D)** Flow cytometry analysis for megakaryocytic CD41a and CD61 and erythroid CD71 and CD235 markers in WAS-siN-WASP *versus* scrambled-shN-WASP cells. The unstained identical plots (left most panels) were used as negative control. **(E)** Summary and statistics of the flow cytometry experiments (n = 3). **(F)** Model depicting the regulation of megakaryopoietic genes by FLI1 *via* WASP/WIP through a dependent and independent mechanism. Upregulation of GATA1 through WASP loss increases FLI1 expression that has a compensatory effect during megakaryocytic differentiation in WAS patients. **P<0.01.

To probe the consequence of N-WASP depletion, we screened and identified a potent siRNA (si1) that effectively (70%) reduced its protein level (**Supplementary Figure 7** **and** **Figures 8A, B**). Depletion of N-WASP by si1 in shWASP-HEL (designated WAS-siN-WASP) cells resulted in a significant decrease in cell proliferation (**Figure 8C**). In contrast to the increased growth rate following WASP knockdown (**Figure 4C**), the lower proliferation of WAS-siN-WASP cells suggests that N-WASP may act as an oncogene or growth promoting factor in erythroid cells (**Figure 8C**). Flow cytometry analysis revealed that while percentage of WAS-siN-WASP cells expressing CD41a and CD71 was reduced, no significant change in the level of CD61 and CD235a was observed (**Figures 8D, E****)**, further demonstrating the importance of N-WASP in megakaryopoiesis.

Next, we used a Wiskostatin [a specific inhibitor of N-WASP (38, 39);], to probe the effect of blocking N-WASP on WASP and FLI1. Wiskostatin treatment (for 24 h) of shWASP-HEL cells downregulated not only N-WASP, but also WASP and FLI1 protein in a dose dependent manner (**Supplementary Figure 8A**). Wiskostatin seems to not effect mRNA expression of these genes (**Supplementary Figure 8B**). Wiskostatin treatment in shWASP-HEL cells resulted in a dose dependent downregulation of CD41a, CD61 as well as CD71 and CD235a (**Supplementary Figure 8C**). Overall, these results revealed that combined depletion of WASP and N-WASP further suppresses megakaryopoiesis, a condition that may exist in WAS patients with severe platelet depletion. We propose that WASP/N-WASP and WIP regulate megakaryopoiesis downstream of FLI1, and this process controlled through WASP-, N-WASP- and WIP-dependent and independent pathways (**Figure 8F**).

## Discussion

In addition to its oncogenic activity, FLI1 is a major player in megakaryopoiesis, although the underlying mechanism is still unknown. Indeed, FLI1 deficiency causes abnormal megakaryocyte development in human and mice (12–16). Here we discovered a direct correlation between FLI1 levels and expression of WASP and its binding partner WIP in leukemic cells. Binding of WASP and WIP is shown critical for normal platelet development, and mutations inhibiting this protein–protein interaction is associated with X-linked thrombocytopenia in WAS patients (28, 40). While the expression of the related gene N-WASP was not regulated by FLI1, its knockdown in leukemic cells was significantly reduced megakaryocytic gene expression. This process was further accelerated when both WASP and N-WASP were depleted in these cells. Moreover, WASP/WIP/N-WASP loss upregulates FLI1 to compensate megakaryocyte deficiency. These results for the first time connect FLI1 to Wiskott–Aldrich Disease and its associated syndromes.

Approximately 89% of patients with the *WAS* gene mutations are classified as exhibiting a severe phenotype, 10% show a mild X-linked thrombocytopenia (XLT) phenotype, and a small number exhibit X-linked neutropenia (3, 41). Thrombocytopenia is also a typical characteristic of FLI1 deficiency in Paris-Trousseau/Jacobsen syndrome (14–16). Mild cytopenia was recently identified in patients having hemizygous mutations in FLI1 and RUNX1 (42). Mutation within WIP was recently identified in a patient exhibiting a severe WAS phenotype, in which the *WAS* gene and its expression remained intact (43). While the incidence of the N-WASP mutation in man has not yet been reported, this report suggests a possible role of this gene in a WAS-related illness. Overall, this study may implicate the *FLI1/WIP/N-WASP* genes in addition to the role of WASP in the microthrombocytopenia associated with WAS.

RNAseq analysis of FLI1 knockdown cells identified defects in several megakaryocyte and platelet formation genes, implicating this TF in many processes of megakaryopoiesis. This study also identified several megakaryocytic genes that are regulated by both FLI1 and WASP. This data further emphasizes the critical role FLI1 plays in megakaryocyte development and suggests a partial role for WASP/WIP in this process. The role of WASP may be restricted to the megakaryocyte development stage and has less impact in late-stage platelet formation, as previously proposed in other systems (44). Knockdown of WASP, WIP, and N-WASP in leukemic cells was found to upregulate FLI1. Higher GATA1 expression in shWASP cells was shown to be responsible for the increased FLI1 expression in the WASP deficient cells. Level of FLI1 upregulation in WAS patients could influence the severity of the diseases due to its compensatory activity (**Figure 8F**). Interestingly, a recently developed FLI1 agonist was capable of inducing megakaryocytic differentiation (17). These compounds may have the potential to partially restore megakaryocytes and platelet defects in WAS patients.

In addition to thrombocytopenia, WAS patients develop other symptoms including immune suppression. Since FLI1 homozygous loss is not viable in man, hemizygous loss of this TF was identified in Paris Trusseau (Jackobsen) syndrome due to chromosome 11q loss or deletions (14–16). Interestingly, in addition to thrombocytopenia, immune deficiency is another known feature of Paris Truseau. Several cases of this disease were identified carrying a combined immune T- and B-cell defect (45). The similarity between Paris Trousseau and WAS syndromes suggests a role for FLI1 in WAS. The reduced expression of common non-megakaryocytic genes detected in both shFLI1 and shWASP knock-down leukemic cells may be responsible for immune suppression or other symptoms of WAS, a notion that requires further investigation.

WASP mutation is associated with RAS pathway mutations in juvenile myelomonocytic leukemia patients, indicating a tumor suppressor activity for this gene (46). Moreover, WASP has been recently identified as a tumor suppressor in T cell lymphoma (30). These results confirm the observation in which WASP depletion in HEL cells caused higher proliferation in culture. The similarity between WASP and WIP growth properties suggests that WIP may also act as a tumor suppressor, a notion that may need further investigation. The CCLE analysis also revealed correlation between the expression of FLI1, WASP, and WIP in hematological malignancies. Interestingly, in contrast to WASP/WIP, knockdown of N-WASP in shWASP-HEL cells suppressed proliferation, suggesting an oncogenic role for this gene during leukemia progression. Since mutation within N-WASP was not yet identified in WAS patients, its loss may confer selective disadvantage to develop WAS syndrome, likely due to its oncogenic ability. The differences between WASP/WIF and N-WASP also suggest a distinct role for these two genes during megakaryopoiesis, as suggested by others (47).

While FLI1 acts as an oncogene, the ability of this TF to induce WASP/WIP, which has a negative growth promoting activity, may counteract its tumor promoting activity. Since FLI1 activation was frequently detected in erythroleukemias (48), it is therefore possible that this TF acts as an oncogene in erythroleukemias, but functions as a tumor suppressor gene in megakaryocytic leukemias. This is consistent with the fact that restoration of FLI1 expression in K562 cells induces megakaryocytic differentiation (17, 49, 50); this dynamic activity may require future investigation.

In summary, a direct correlation between FLI1 and WAS/WIP expression led to the identification of these two genes as a direct regulatory target for FLI1. ShRNA mediated knockdown of WASP and WIP resulted in inhibition of the classical megakaryocytic differentiation markers CD41 and CD61 as well as other genes. These results suggested that FLI1 may induce megakaryocytic differentiation in part through WAS and WIP. This study also raised the possibility that in addition to WASP, WIP and N-WASP, FLI1 may be involved in Wiskott–Aldrich Disease.

## Data Availability Statement

The original contributions presented in the study are publicly available. This data can be found here: https://www.ncbi.nlm.nih.gov/bioproject/?term=PRJNA682304.

## Author Contributions

CW, BG, PK, WL, AH, YL, XH, and EZ contributed to the conception, design of the study, as well as data acquisition and interpretation. PK provided sequencing data, and KS, BG, and YL were involved in data and statistical analysis. CW drafted the manuscript. YB-D, XH, PK, YL, and EZ reviewed the manuscript critically. YB-D supervised, conceived, and designed the study. All authors contributed to the article and approved the submitted version.

## Funding

This study was supported by research grants from the Natural National Science Foundation of China (U1812403, 21867009 and 81872772, 81960546), the Science and Technology Department of Guizhou Province innovation and project grants (QKHPTRC[2019]5627 and QKHJC[2018]1409) and the 100 Leading Talents of Guizhou Province to YB-D, YL, and XH.

## Conflict of Interest

The authors declare that the research was conducted in the absence of any commercial or financial relationships that could be construed as a potential conflict of interest.
