# Supplementary material for: FLI1 Induces Megakaryopoiesis Gene Expression Through WAS/WIP-Dependent and Independent Mechanisms; Implications for Wiskott-Aldrich Syndrome
Source: Front Immunol. 2021 Feb 26;12:607836. doi: 10.3389/fimmu.2021.607836 (PMC7953068; doi:10.3389/fimmu.2021.607836)
Supplement: Supplementary file 1 [file DataSheet_1.pdf]

Gene	Sequences
WASA	5’-TATCCAGAGACCACAGGGGGCTCGCTCTGCGATTTATGGTGCGAGGAGGACCAGACACCCCCTGTGCCCGTCCTACTCCTGCAGATCAAACTCACAGCCACACT TTTCGGAATGAGAAACTGGACAGTAAATGAACGCAAGAACCATGGTGCCCAGGCAGAGAACCCCGCTGGGTTTGCTACGAAGGTTGCGGTGGAGGCGCCCAGTGC ACCAGGCGCCAAGGCCCGGTATGTGACCTCACTAACCTCCGCCAGCGCGCACCCTGTCCTGCCCCTTCTCGGGCCAACAACCCCACACGACGCAGCGGTGTGAG AACCAGCAACCGGAACCGCAAAAGCACGGCTTTCCCGGAGCCCGGGCTCTGGACTGGCACCCACTGCGCATGCTCCAGAGCGGCGACCAATGGGAGCCCGGCG CGCAGGGACGGAGGCGGGCCCGGGCCGCTACCGAGAAGCACAGGGACCCGACTTCAGTCATCGACCAATCAGAGCTCACAGCCCCCCACACCCCCTCCCCCCC GGCCCTGAGCCAATTGCCAGCTCGTGTGCGGGAGGGCCGGGTCAAGACTAAGTC-3’
WASB	5’-GCTCTGCGATTTATGGTGCGAGGAGGACCAGACACCCCCTGTGCCCGTCCTACTCCTGCAGATCAAACTCACAGCCACACTTTTCGGAATGAGAAACTGGACAG TAAATGAACGCAAGAACCATGGTGCCCAGGCAGAGAACCCCGCTGGGTTTGCTACGAAGGTTGCGGTGGAGGCGCCCAGTGCACCAGGCGCCAAGGCCCGGTAT GTGACCTCACTAACCTCCGCCAGCGCGCACCCTGTCCTGCCCCTTCTCGGGCCAACAACCCCACACGACGCAGCGGTGTGAGAACCAGCAACCGGAACCGCAAA AGCACGGCTTTCCCGGAGCCCGGGCTCTGGACTGGCACCCACTGCGCATGCTCCAGAGCGGCGACCAATGGGAGCCCGGCGCGCAGGGACGGAGGCGGGCCC GGGCCGCTACCGAGAAGCACAGGGACCCGACTTCAGTCATCGACCAATCAGAGCTCACAGCCCCCCACACCCCCTCCCCCCCGGCCCTGAGCCAATTGCCAGCT CGTGTGCGGGAGGGCCGGGTCAAGACTAAGTCAAAGGAGGAGAGGG-3’
WASC	5’-TGCGCCTGGAAAACTGAGGAAAGTGTTTATTCCCTTACACACGCTTCGCCAAAGGCCCTGGCACGTAGGGCTTTTTCACTGCTGCGAACGTTAGACCGGCCTGT GATAAGGAGTACAATTAATCTAACTTTGGTATACTATATCCAGAGACCACAGGGGGCTCGCTCTGCGATTTATGGTGCGAGGAGGACCAGACACCCCCTGTGCCCG TCCTACTCCTGCAGATCAAACTCACAGCCACACTTTTCGGAATGAGAAACTGGACAGTAAATGAACGCAAGAACCATGGTGCCCAGGCAGAGAACCCCGCTGGGTT TGCTACGAAGGTTGCGGTGGAGGCGCCCAGTGCACCAGGCGCCAAGGCCCGGTATGTGACCTCACTAACCTCCGCCAGCGCGCACCCTGTCCTGCCCCTTCTCG GGCCAACAACCCCACACGACGCAGCGGTGTGAGAACCAGCAACCGGAACCGCAAAAGCACGGCTTTCCCGGAGCCCGGGCTCTGGACTGGCACCCACTGCGCAT GCTCCAGAGCGGCGACCAATGGGAGCCCGGCGCGCAGGGACGGAGGCGGGCCCGGGCCGCTACCGAGAAGCACAGGGACCCGACTTCAGTCATCGACCAATCA GAGCTCACAGCCCCCCACACCCCCTCCCCCCCGGCCCTGAGCCAATTGCCAGCTCGTGTGCGGGAGGGCCGGGTCAAGACTAAGTCAAAGGAGGAGAGGGCAA CGCGGTGGGC -3’
N-WASP	5’-ACGCAACAAAAATATGTTTAATGTGGAGAACAGTGAGATGCATATTTGATAATTCACTGCAGTCTGCATCATCATCTCTATATTCCAAAATTTCGTCTTAAGCCGTTT AGTATTTAACTTACAGAAGAAAGGGGGTTATCTTAGATAACTTTTAATATCCTAAGGTATTTTCCAATTCACGATACAGGGAGAAAAGAAATGTGTCCAGGCCCTTAGA AGAAAATACTTTGAATTTAAATGTGACACATACATTAAACCCGCGTGACACGCCAGGCAGGCGGTGGAAGTCAAGTGCATTTGTTTTTGTTTCATCCACCAGGGCGA TCTCTTTCACAATTCTGGGAGAGGCCTGTTTGTTCCTCCACTCCCACAGCTGAAGTTTGGGACAAAGTTTGAGAAAATCCCAGGCACCTGCGGCTCCTATCGAAGCC GGCGAGGAGCACAGGGTAGAGCGTCTCTTCCCAGTTTTCAGTCTTCAGATCGCAGGCCTCTCGGACCCGCAGAGCGAGCTTCACGCTGCAGCGCTTGAGAAATAA GAGCTATTCCAAGGAGGGGCCCTGGGTCCTCACTGACGCTTCGCAAAGGTGTACGTCCGCCCAGGGAAGGCCGGCGGCGCAGCGACGGCGAGGAGTCCCCATC CATCTTCTCTTCAAGCAGCAGTAGCTGGGTTCGGGTACCCAACCGACGAGCCGAGACGCCAGAGGCCAGAGTACGAAGTTGGAAGCCTGCGCCCCAAGCCAACC GGGATTCCACTTAGCTCCGCGCGGTCCGCGCCTCGGCCGGAAGGGGGCGTGGCTTCGGGCCCGCGCCGCGAGGCCCGCCGCCGAGTGGGGGGTTTCTCCCG GGCCGCAGCCGCGCGTTTCCGGCGCGCTCCCCCGCGCCCTCCGTCCTGTGGTCTCGCCCGCCCCCGCTGCCATGTTGGATTGTGCGGCCGCCGCCGCCGCTGC GGGAGGG-3’

Supplementary Table 1 | Sequence of promoter fragments used for cloning.

**WIP shRNA**	
WIP-sh1	5'-CCTCCACCATCAACATCTATTTCAAGAGAATAGATGTTGATGGTGGAGGTTTTTT-3'
WIP-sh2	5'-CCAATACTGGACAAACCTAAATTCAAGAGATTTAGGTTTGTCCAGTATTGGTTTTTT3'
WIP-sh3	5'-CATTCAATCAAGTCCGCACAATTCAAGAGATTGTGCGGACTTGATTGAATGTTTTTT-3'
**N-WASP shRNA**	
N-WASP-sh1	5'-GCAAGAAATGTGTGACTATTTCAAGAGAATAGTCACACATTTCTTGCTTTTTT-3'
N-WASP-sh2	5'-GGGAACAAGAGCTATACAATTCAAGAGATTGTATAGCTCTTGTTCCCTTTTTT-3'
N-WASP-sh3	5'-GCAGATATAGGAACACCAATTCAAGAGATTGGTGTTCCTATATCTGCTTTTTT-3'
**WASP shRNA**	
WASP-sh1	5'-CGAGACCTCTAAACTTATCTATTCAAGAGATAGATAAGTTTAGAGGTCTCGTTTTTT-3'
**N-WASP siRNA**	
N-WASP-si1	5'-GCAAGAAAUGUGUGACUAUTTAUAGUCACACAUUUCUUGCTT-3'
N-WASP-si12	5'-GGAACUGUAUGUGGUCAAATTUUUGACCACAUACAGUUCCTT-3'
N-WASP-si13	5'-GCUGAUGGCCAAGAGUCUATTUAGACUCUUGGCCAUCAGCTT-3'
**GATA1 siRNA**	
GATA1-si1	5'-CCCUGCCUCAACUGU GUGUTTACACACAGUUGAGGCAGGGTT-3'
GATA1-si2	5-CUCGAAACCG CAAGGCACUTTGAUGCCUUGCGGUUUCGAGTT-3'
GATA1-si13	5-GCGCC UGAUU GUCAG UAAATTUUUAC UGACAAUCAG GCGCTT-3'

Supplementary Table 2 | ShRNA and siRNA sequences.

Genes	Sense	Antisense
β-actin	CATGTACGTTGCTATCCAGGC	CTCCTTAATGTCACGCACGAT
GAPDH	AGAAGGCTGGGGCTCATTTG	AGGGGCCATCCACAGTCTTC
WAS	TATTGGCGTTGAAAGGGGCA	AGCAGCCGTTGTTCTGTGAA
N-WASP	AGGGTCACCAACGTGGGGTC	TGCAGGCCAAAGTCAGAGTC
WIP	CCGGGAGAAGTTTCCCAGAA	TCCCAGCCTGCTCTGTCTTA
FLI1	CCAACGAGAGGAGAGTCATCG	TTCCGTGTTGTAGAGGGTGGT
GATA1	CTGTCCCCAATAGTGCTTATGG	GAATAGGCTGCTGAATTGAGGG
Primers used for cloning		
WASA	GGGGTACCTATCCAGAGACCACAGGGGG	CCGCTCGAGGACTTAGTCTTGACCCGGCC
WASB	GGGGTACCGCTCTGCGATTTATGGTGCG	CCGCTCGAGTGCCCTCTCCTCCTTTGACT
WASC	GGGGTACCTGCGCCTGGAAAACTGAGGAAAG	CCGCTCGAGGCCCACCGCGTTGCCCTCTCCT
Primers used for chromatin immunoprecipitation analysis		
WAS-FBS	CGAAGGTTGCGGTGGAGGCGCCCAG	CCGTCCCTGCGCGCCGGGCTCC
WAS-NC	CCAAAGGCCCTGGCACGTAG	CCTCCTCGCACCATAAATCGC
WIP-BST	TCAGATGCGTGTGGACCTTG	TCCACCTTCTTGCGGCTG
WIP-NC	GCAGAGCCCCATTGCTGG	GGGAACTTCGCCGAGATGT

Supplementary Table 3 | Primers for real time PCR.

Gene symbol	Gene Name	GO Term	Scrambled-FPKM	shFLI1-FPKM	shWASP-FPKM
MEIS1	Homeobox protein Meis1	GO:0035855	20.2114	7.20889	20.0011
GP1BA	Platelet glycoprotein Ib alpha chain	GO:0045652	31.8054	7.25409	57.1396
FLI1	Friend leukemia integration 1 transcription factor	GO:0035855	101.17	12.5119	120.287
AGO1	Protein argonaute-1	GO:0045652	25.1843	18.2496	22.5069
AGO3	Protein argonaute-3	GO:0045652	25.1843	18.2496	22.5069
KAT2B	Histone acetyltransferase KAT2B	GO:0045652	16.0469	21.799	11.1721
WDR5	WD repeat-containing protein 5	GO:0045652	21.12	22.6915	25.6715
PTPN6	Tyrosine-protein phosphatase non-receptor type 6	GO:0035855	95.3272	24.481	71.9079
KMT2A	Histone-lysine N-methyltransferase 2A	GO:0045652	27.8565	26.0746	24.8291
SP3	Transcription factor Sp3	GO:0030219	23.5971	26.2309	22.2199
KIT	Mast/stem cell growth factor receptor Kit	GO:0035855	52.2071	26.4969	37.7547
GABPA	GA-binding protein alpha chain	GO:0045653	23.0808	26.975	24.1974
SH2B3	SH2B adapter protein 3	GO:0035855	47.8562	27.5587	54.3642
SRF	Serum response factor	GO:0035855	29.0867	28.6268	27.718
ABI1	Abl interactor 1	GO:0035855	29.0495	28.9692	31.1022
PRKCQ	Protein kinase C theta type	GO:0045652	36.7414	35.1422	31.0755
SIN3A	Paired amphipathic helix protein Sin3a	GO:0045652	44.8683	39.6871	51.3388
MTURN	Maturin	GO:0045654	49.6206	41.45	36.1856
CBFB	Core-binding factor subunit beta	GO:0045652	60.775	42.4897	50.0507
PIP4K2A	Phosphatidylinositol 5-phosphate 4-kinase type-2 alpha	GO:0035855	51.8152	48.7868	48.9073
WASF2	Wiskott-Aldrich syndrome protein family member 2	GO:0035855	48.6722	49.1689	59.0909
ZNF385A	Zinc finger protein 385A	GO:0035855	91.0255	49.2589	137.639
KMT2D	Histone-lysine N-methyltransferase 2D	GO:0045652	46.9837	49.6147	43.6074
MEF2C	Myocyte-specific enhancer factor 2C	GO:0045652	61.2964	55.3727	85.4285
PTPN11	Tyrosine-protein phosphatase non-receptor type 11	GO:0035855	73.1857	61.5537	75.0636
MOV10	Helicase MOV-10	GO:0045652	26.8755	64.8313	25.5154
RUNX1	Runt-related transcription factor 1	GO:0045652	121.63	70.1504	127.748
GATA2	Endothelial transcription factor GATA-2	GO:0045654	164.774	73.2839	133.222
FAXDC2	Fatty acid hydroxylase domain-containing protein 2	GO:0045654	5.65197	76.9597	8.50001
CIB1	Calcium and integrin-binding protein 1	GO:0045653	61.6796	82.7761	64.7237
PITHD1	PITH domain-containing protein 1	GO:0045654	63.6714	84.5133	62.2201
TAL1	T-cell acute lymphocytic leukemia protein 1	GO:0030219	84.163	88.9375	87.6264

ASH2L	Set1/Ash2 histone methyltransferase complex subunit ASH2	GO:0045652	60.6467	89.0361	66.6531
ITGA2B	Integrin alpha-IIb	GO:0045652	280.562	105.002	307.612
HDAC1	Histone deacetylase 1	GO:0045652	99.8049	119.359	113.116
DPY30	Protein dpy-30 homolog	GO:0045652	111.527	120.434	109.886
PRMT1	Protein arginine N-methyltransferase 1	GO:0045653	180.979	140.569	202.131
EIF6	Eukaryotic translation initiation factor 6	GO:0045652	215.767	224.131	233.931
GATA1	Erythroid transcription factor	GO:0045652	174.872	224.536	190.436
NFE2	Transcription factor NF-E2 45 kDa subunit	GO:0045652	212.272	270.685	167.023
TESC	Calcineurin B homologous protein 3	GO:0045654	146.672	426.445	166.488
H3-3A	Histone H3.3	GO:0045652	558.896	548.688	634.619
HMGB2	High mobility group protein B2	GO:0045654	547.456	957.481	596.232

Supplementary Table 4 | FLI1 regulates megakaryocytic differentiation in part through WASP/WIP. Transcription of selected megakaryocytic genes in shFLI1 and shWASP expressing HEL cells. Scrambled vector used as control.

Gene symbol	Gene	Scrambled-FPKM	shFLI1-FPKM	shWASP-FPKM
GATA1	Erythroid transcription factor	174.872	224.536	190.436
CD36	Platelet glycoprotein 4	12.4724	105.754	12.9078
PRKDC	DNA-dependent protein kinase catalytic subunit	82.2529	93.2822	87.9521
MYH9	Myosin-9	99.3428	90.917	92.2699
TAL1	T-cell acute lymphocytic leukemia protein 1	84.163	88.9375	87.6264
CIB1	Calcium and integrin-binding protein 1	61.6796	82.7761	64.7237
WDR1	WD repeat-containing protein 1	106.981	66.3677	114.916
PTPN11	Tyrosine-protein phosphatase non-receptor type 11	73.1857	61.5537	75.0636
MEF2C	Myocyte-specific enhancer factor 2C	61.2964	55.3727	85.4285
ZNF385A	Zinc finger protein 385A	91.0255	49.2589	137.639
CLEC1B	C-type lectin domain family 1 member B	47.2202	33.8467	57.4535
SRF	Serum response factor	29.0867	28.6268	27.718
ACTN1	Alpha-actinin-1	53.3006	28.6094	55.7117
CASP3	Caspase-3	38.6794	28.0223	26.2279
VPS33A	Vacuolar protein sorting-associated protein 33A	19.0754	24.7791	21.2729
NBEAL2	Neurobeachin-like protein 2	26.9271	24.6248	29.1649
PTPN6	Tyrosine-protein phosphatase non-receptor type 6	95.3272	24.481	71.9079
MPL	Thrombopoietin receptor	39.4804	7.92115	40.209

Supplementary Table 5 | Regulation of platelet formation genes by FLI1 and WASP. Transcription of selected platelet formation genes in shFLI1 (shFLI1-FPKM) and shWASP (shWAS-FPKM) expressing HEL cells. Scrambled vector used as control.

Gene	Scrambled	shFLI1	shWASP
WAS	47.5489	19.4628	6.1323
AK6,TAF9	56.7895	17.761	8.62721
CAPG	64.3893	4.34325	23.9486
DHRS2	26.2336	4.23597	7.43023
IFI27L2	31.3118	7.98431	7.16441
HDC	103.905	4.96574	12.414
PFDN6	71.5895	10.2522	11.0252
TPSAB1	56.2207	6.1269	6.82084

Supplementary Table 6 | Regulation of non-megakaryocytic genes by FLI1 and WASP. Expression of genes downregulated strongly in shFLI1 (shFLI1-FPKM) and shWASP (shWAS-FPKM) expressing HEL cells. Scrambled vector used as control.
