# Supplementary material for: FLI1 Induces Megakaryopoiesis Gene Expression Through WAS/WIP-Dependent and Independent Mechanisms; Implications for Wiskott-Aldrich Syndrome
Source: Front Immunol. 2021 Feb 26;12:607836. doi: 10.3389/fimmu.2021.607836 (PMC7953068; doi:10.3389/fimmu.2021.607836)
Supplement: Supplementary file 2 [file DataSheet_2.pdf]

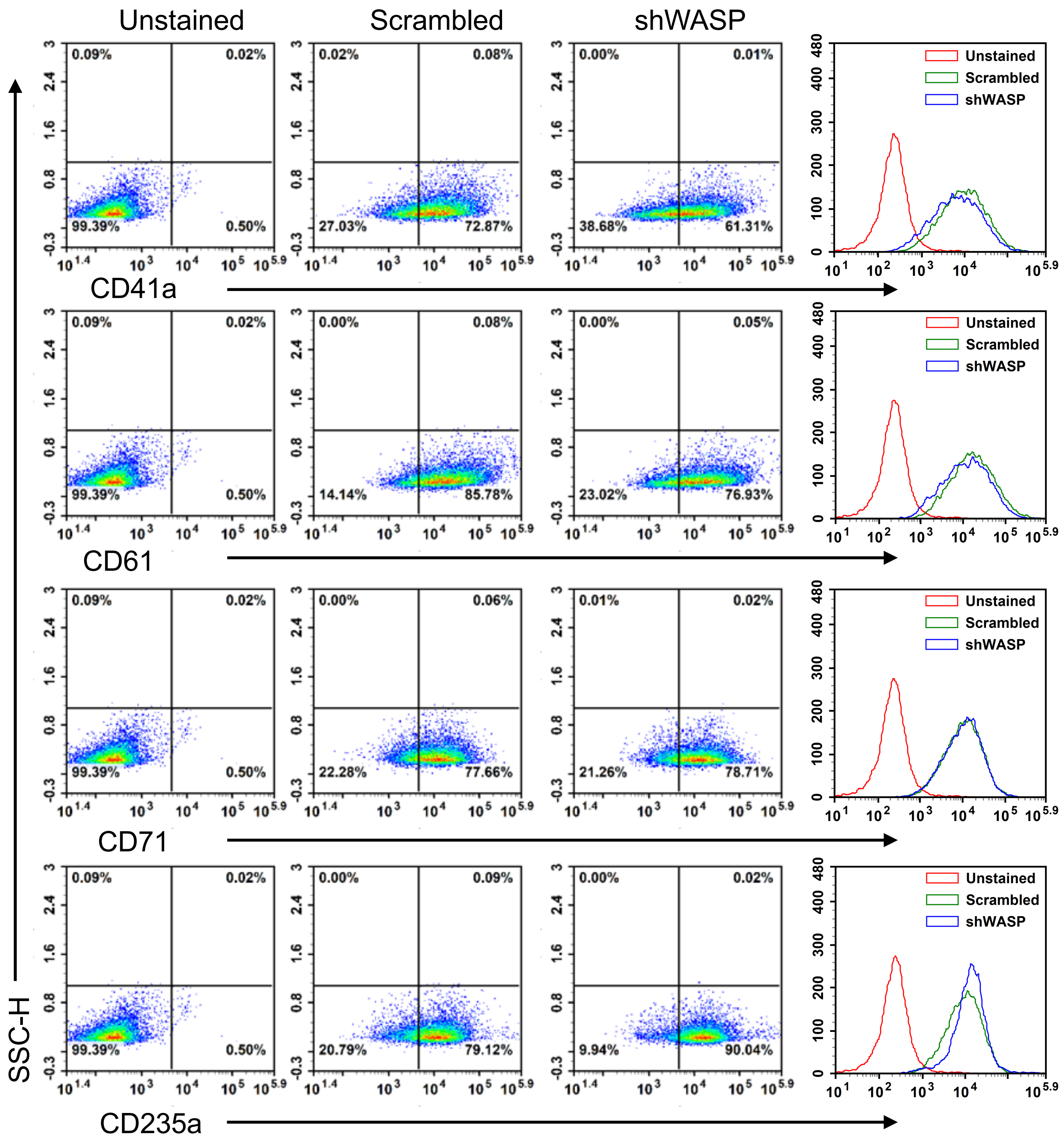

Figure S1. WASP knockdown reduced the expression of megakaryocytic CD41a and CD61 genes. Flow cytometry analysis of shWASP-HEL versus scrambled-HEL cells for expression of CD41a, CD61, CD71 and CD235a cell surface markers.

.

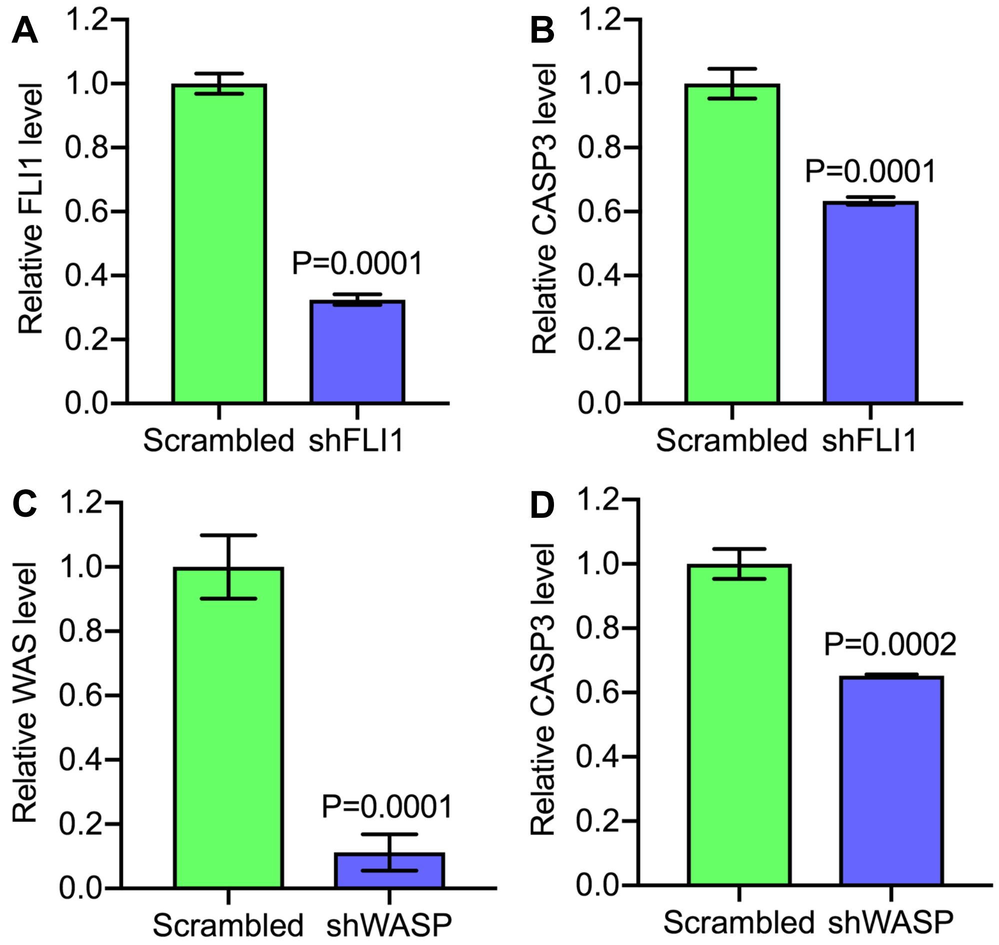

Figure S2: Downregulation of platelet gene CASP3 in shFLI1 shWASP cells. (A-B) Knockdown of FLI1 (A) in sh-FLI1 relative to scrambled control HEL cells resulted in down regulation of Platelet specific gene CASP3, by Q-RT-PCR. (C-D) Knockdown of *WAS* (C) in sh-WASP relative to scrambled control HEL cells also resulted in down regulation of CASP3, by Q-RT-PCR.

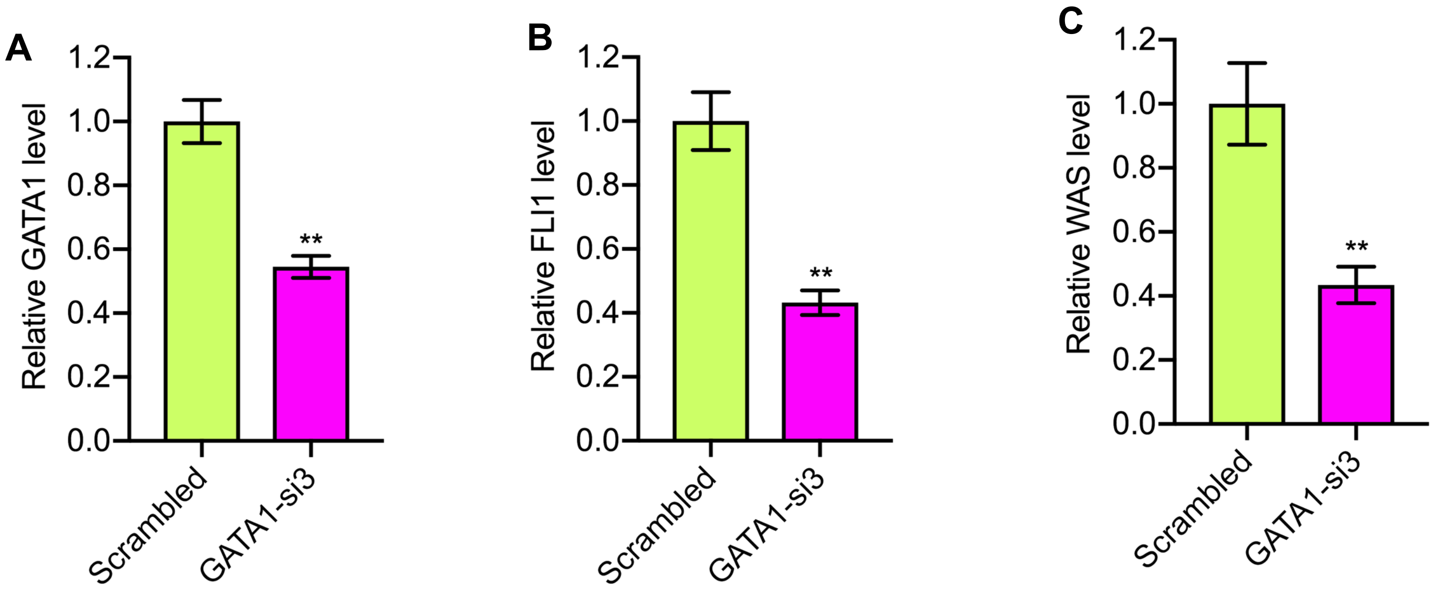

Figure S3: FLI1 expression is regulated by GATA1. (A-C) Downreguation of GATA1 (A) by GATA1-si3 in shWASP cells. Resulted in downregulation of FLI1 (B) and WAS (C).

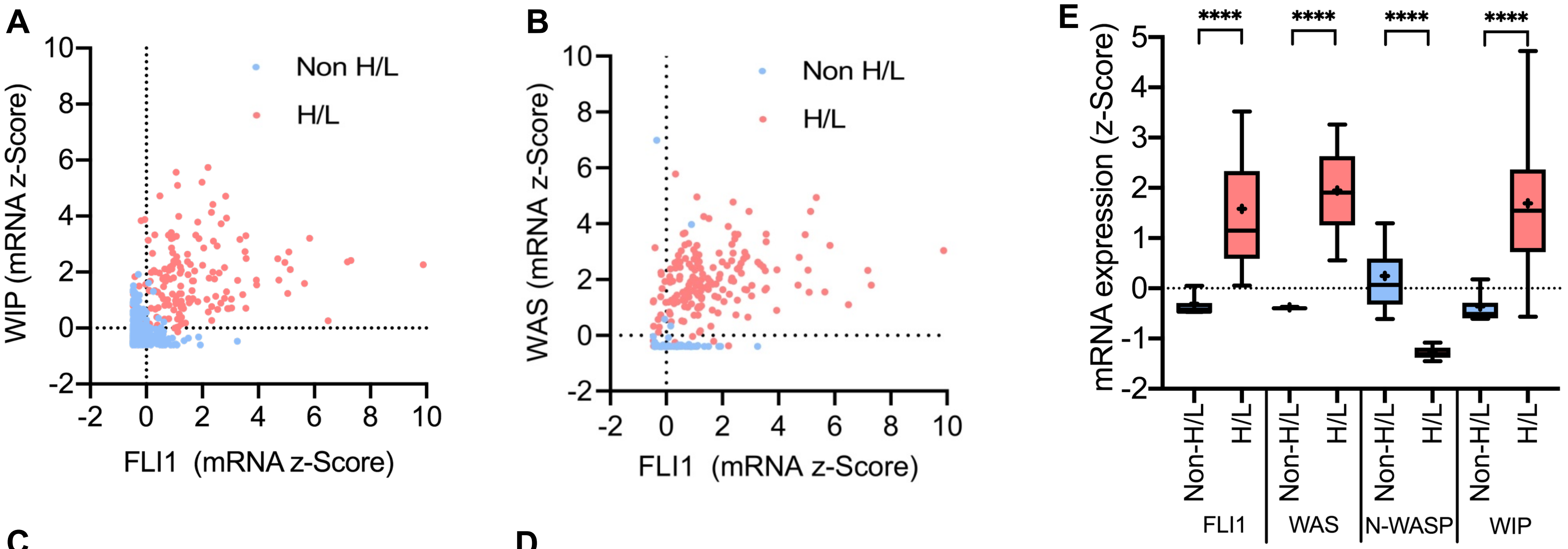

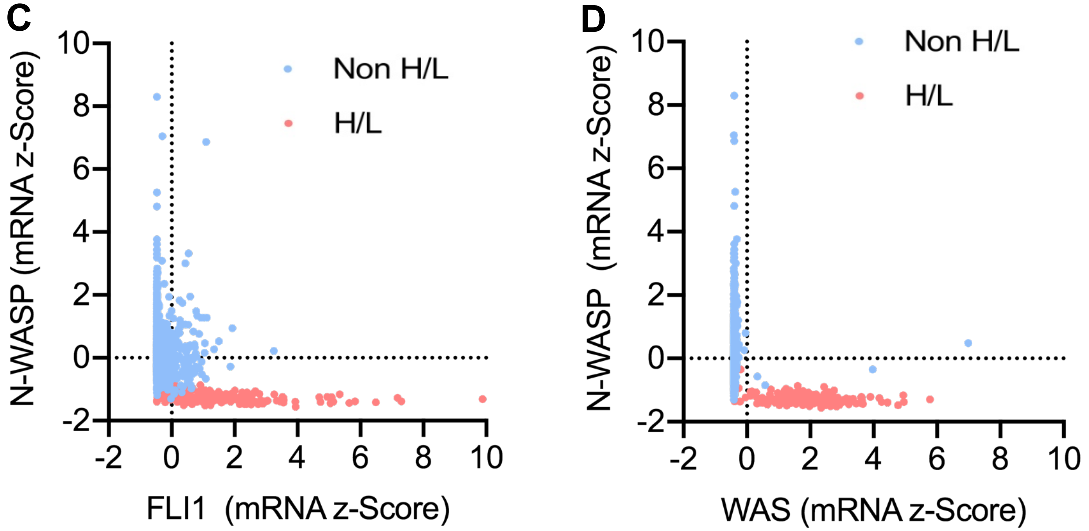

F

		FLI1 vs WAS	WAS vs N-WASP	FLI1 vs N-WASP	FLI1 vs WIP
All CCLE Cells	Spearman r	0.2757	-0.3195	-0.4497	0.6355
	P (two-tailed)	<0.0001	<0.0001	<0.0001	<0.0001
H/L	Spearman r	0.3234	-0.252	-0.3165	0.2673
	P (two-tailed)	<0.0001	<0.0005	<0.0001	0.0005
Non H/L	Spearman r	-0.1551	0.1296	-0.1308	0.4216
	P (two-tailed)	<0.0001	<0.0001	<0.0001	<0.0001

Figure S4: Correlations between WAS, WIP, N-WASP and FLI1 in hematological and non-hematological malignancies. (A-B) There is a significant direct correlation between the expression of WAS/FLI1 (A) and WIP/FLI1 (B) in hematopoietic and lymphoid (H/L, red; non H/L, blue) cancer cell lines. Such correlation only seen in non-hematological (non H/L) malignancies between N-WASP/FLI1 (C) and N-WASP/WAS (D). (E) Summary of expression of FLI1, WAS, N-WASP and WIP expression in H/L and non H/L cancer cell lines.****P=<0.0001. (F) Table of Spearman r and p values for the indicated groups.

----ACGCAACAAAAATATGTTTAATGTGGAGAACAGTGAGATGCATATTTGATAATTCA
CTGCAGTCTGCATCATCATCTCTATATTCCAAAATTTCGTCTTAAGCCGTTTAGTATTTA
ACTTACAGAAGAAAGGGGGTTATCTTAGATAACTTTTAATATCCTAAGGTATTTTCCAAT
TCACGATACAGGGAGAAAAGAAATGTGTCCAGGCCCTTAGAAGAAAATACTTTGAATTTA
AATGTGACACATACATTAAACCCGCGTGACACGCCAGGCAGGCGGTGGAAGTCAAGTGCA
TTTGTTTTTGTTTCATCCACCAGGGCGATCTCTTTCACAATTCTGGGAGAGGCCTGTTTG
TTCCTCCACTCCCACAGCTGAAGTTTGGGACAAAGTTTGAGAAAATCCCAGGCACCTGCG
GCTCCTATCGAAGCCGGCGAGGAGCACAGGGTAGAGCGTCTCTTCCCAGTTTTCAGTCTT
CAGATCGCAGGCCTCTCGGACCCGCAGAGCGAGCTTCACGCTGCAGCGCTTGAGAAATAA
GAGCTATTCCAAGGAGGGGCCCTGGGTCCTCACTGACGCTTCGCAAAGGTGTACGTCCGC
CCAGGGAAGGCCGGCGGCGCAGCGACGGCGAGGAGTCCCCATCCATCTTCTCTTCAAGCA
GCAGTAGCTGGGTTCGGGTACCCAACCGACGAGCCGAGACGCCAGAGGCCAGAGTACGAA
GTTGGAAGCCTGCGCCCCAAGCCAACCGGGATTCCACTTAGCTCCGCGCGGTCCGCGCCT
FLI1 binding site → CGG**CCGGAAGG**GGGCGTGGCTTCGGGCCCGCGCCGCGAGGCCCGCCGCCGAGTGGGGGG
TTTCTCCCGGGCCGCAGCCGCGCGTTTCCGGCGCGCTCCCCCGCGCCCTCCGTCCTGTGG
TCTCGCCCGCCCCCGCTGCC**ATGTTGGATTGTGCGGCCGCCGCCGCCGCTGCGGGAGGG**-
+1

Figure S5: Promoter of the *N-WASP* gene. Above shows the promoter of human N-WASP and localization of FLI1 binding site within -129 to -137 residue.

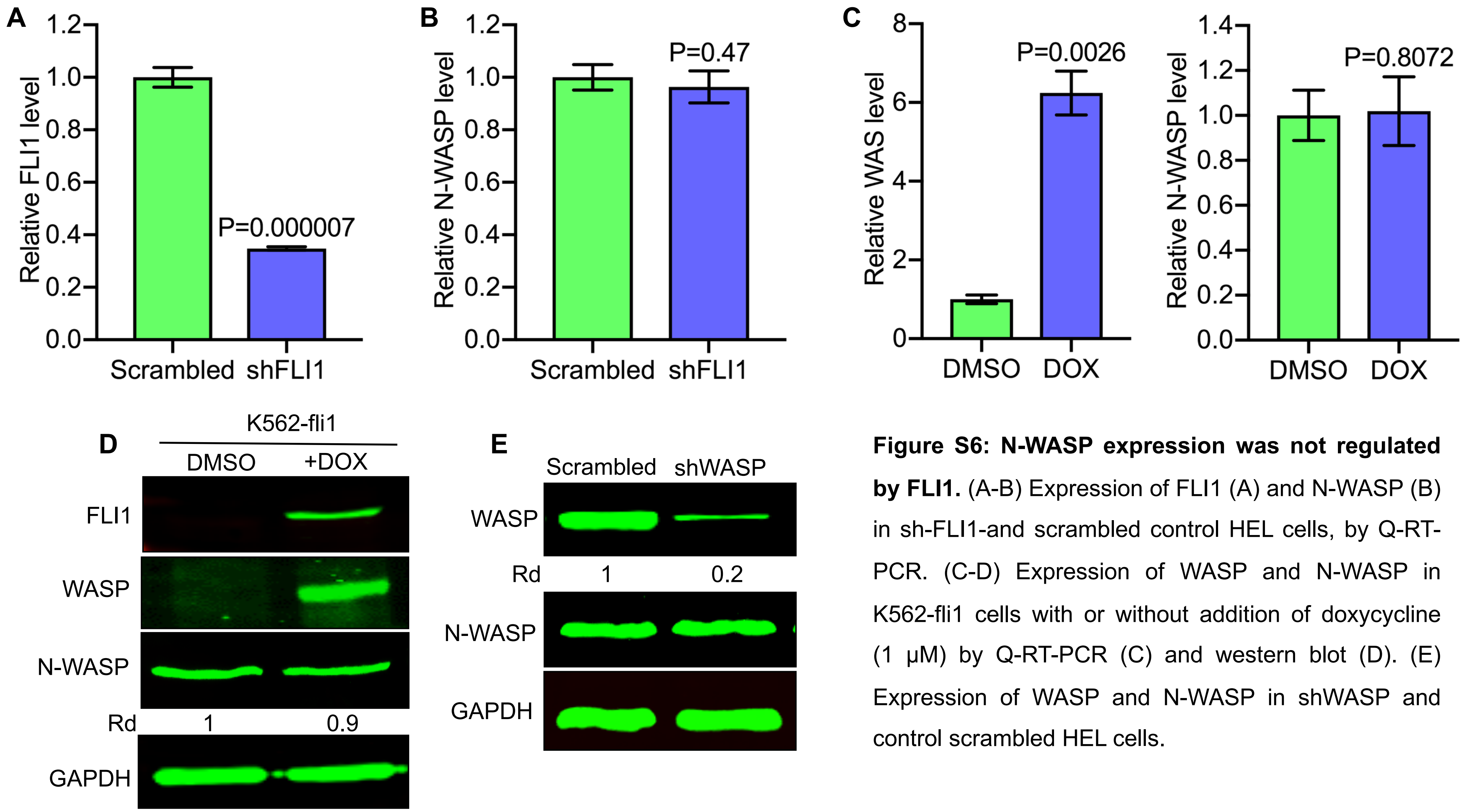

Figure S6: N-WASP expression was not regulated by FLI1. (A-B) Expression of FLI1 (A) and N-WASP (B) in sh-FLI1-and scrambled control HEL cells, by Q-RT-PCR. (C-D) Expression of WASP and N-WASP in K562-fli1 cells with or without addition of doxycycline (1 μM) by Q-RT-PCR (C) and western blot (D). (E) Expression of WASP and N-WASP in shWASP and control scrambled HEL cells.

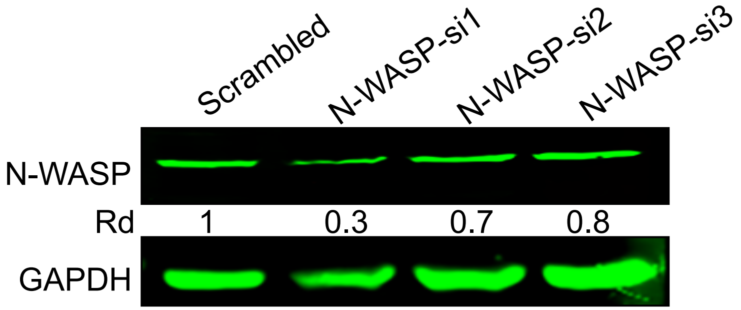

Figure S7: SiRNA mediated knockdown of N-WASP in shWASP-HEL cells. Three siRNAs (si1-si3) were used to transfect shWASP-HEL cells. Scrambles siRNA was used as control. Rd: relative density.

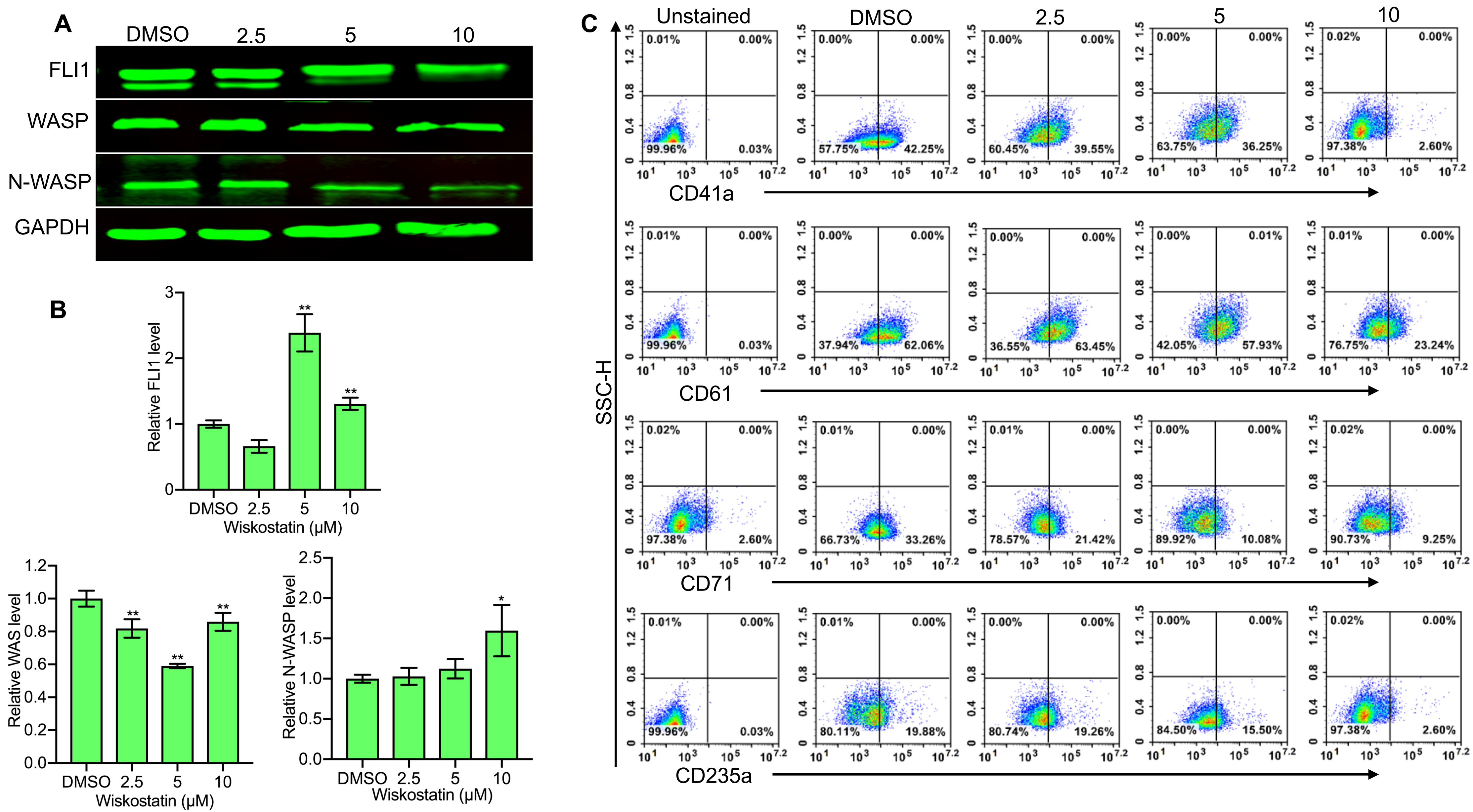

Figure S8: Wiskostatin downregulates at post-transcriptonal level expression of various genes. (A-B) ShWASP-HEL cells were treated with indicated concentrations of Wiskostatin (μM) for 24 hours and expression of the FLI1, WASP and N-WASP genes was determined by western blot (A) or Q-RT-PCR (B). (C) Flow cytometry analysis of shWASP-HEL cells after treatment with Wiskostatin for 18 hours for expression of CD41a, CD61, CD71 and CD235a.
